# Utopia, theodicy, and ritual: East Asian perspectives

**DOI:** 10.1080/0048721X.2024.2362069

**Published:** 2024-06-27

**Authors:** Aike P. Rots

**Affiliations:** Department of Culture Studies and Oriental Languages (IKOS), University of Oslo, Oslo, Norway

**Keywords:** Pollution, ritual theory, Tenrikyō

## Abstract

This response argues that ‘utopia' and ‘utopianism' are useful conceptual tools for analysing and comparing social practices in different historical and geographical contexts. It also suggests that the theoretical interventions put forward in this special issue can be developed further by bringing them in dialogue with cases and theories from East Asia. The response introduces the example of Tenri City in Japan, conceived in the early postwar period as a physical realisation of the Tenrikyō Church’s utopian vision, which today comes across as an outdated urban planning project. It asks how groups such as Tenrikyō reconcile their utopian promises with the inherent imperfections that characterise any real-world social project, focusing on two key themes: theodicy and ritual action. The insights from this approach contribute to broader discussions in the study of religion\s, underscoring the interplay between temporal imagination, materiality, and everyday ritual practices in sustaining community life and social identity.

## Finding new metalanguage

In the last twenty years, much has been written about the usefulness, or lack thereof, of ‘religion’ as a universal category. Numerous scholars have historicised the category, discussed its historical formation and genealogy, and analysed the ways in which it has been adopted and adapted in a variety of colonial and other non-Western contexts (Asad 2003; Josephson 2012; Masuzawa 2005; Nongbri 2013; Smith 1998). As a result, few scholars of religion these days use terms like ‘religious' and ‘religiosity’ non-reflexively, and few still perceive ‘religion’ as a *sui generis* category describing a foundational aspect of human thought and social practice (McCutcheon 1997). ‘Religion’ remains meaningful as an emic concept used for identity formation and governance in nation-states around the globe, as well as in international politics. However, it has become problematic as a second-order, etic concept that can be employed to analyse and compare disparate practices across time and space, because it is configured and demarcated so differently in different contexts. In other words, the critical turn in the study of religion has led to some profound insights into the political embeddedness of religion-making and the discipline's own colonial history (Dressler and Mandair 2011), but it has also led to the loss of metalanguage, as discussed by Kirsch and Rota in their introduction to this special issue. It appears as if, as a discipline, we are at a loss for words. That is, we still compare ideas and practices in different historical periods and geographical locations, if only implicitly – how could we not, when comparison constitutes our discipline's foundational raison d’être – but we often struggle to find the right conceptual tools to engage in more explicitly comparative analyses.

As Oliver Freiberger has argued, like other disciplines, the academic study of religion needs metalanguage in order to be able to engage in meaningful comparison (Freiberger 2019). That does not mean, however, that we can simply reinstate ‘religion’ as our disciplinary master category, once again turning the scholar of religion into the arbiter of what does or does not count as religious (which, as some of the ‘implicit religion’ scholarship illustrates, can be pretty much anything). Just as critical heritage scholars acknowledge that ‘heritage’ is not an intrinsic property of things, but a category of governance and tool for nation-building that is attributed selectively to certain designated places and practices (Harrison 2012; Lowenthal 1998), so scholars of religion need to pay attention to the ways in which powerful institutional and state actors demarcate ‘religion’ and use it for purposes of differentiation. In this regard, ‘religion’ and ‘heritage’ are similar: both are successfully universalised, both mean different things in different contexts, both are powerful categories of governance employed by state actors, and neither describes any *intrinsic* qualities of particular places and practices. Because of its political embeddedness, therefore, ‘religion’ can and should no longer be the basis for comparing – unless, of course, when comparing legal, discursive, and material processes of religion-making in different contexts. It is a powerful emic term, but problematic as an etic term.

So what metalanguage *can* we use? What other comparative, theoretical, second-order concepts do we have at our disposal, if not ‘religion’? There are of course various generic concepts that have a long history within the field, each with its own legacy, connotations, and pros and cons: ‘ritual’, ‘symbol’, ‘sacred’, ‘doctrine’, ‘faith’, ‘temple’, ‘pilgrimage’, ‘priest’, and more. To varying degrees, these terms remain useful as heuristic tools, even if the phenomena to which they refer differ considerably from place to place. Other terms, such as ‘mediation’, ‘affect’, ‘embodiment’, ‘gender’, ‘materiality’, and ‘marketplace’, have become part of the religious studies metalanguage more recently, but are already proving useful for analysing phenomena and developments across time and space in new ways, providing important new insights. And yet other terms, such as ‘corporation’, ‘more-than-human’, and ‘Anthropocene’, have entered the stage very recently, but have the potential to change the way we think and write about worship practices and religious institutions. So, arguably, has ‘utopia’ – which is not a new term per se, but which, as the editors of this special issue point out, has a peripheral status within religious studies. Putting ‘utopia’ at the centre of our analysis may help us see phenomena in a new light and see connections and similarities between ideas and practices in different places, regardless of whether or not they are classified as religious. Thus, I agree with Anja Kirsch that the notion of (vernacular) utopia can be a helpful tool for overcoming problematic conceptual binaries. As such, ‘utopia’ and ‘utopianism’ have the same potential as a term like corporation (McLaughlin et al. 2020): they can help us acknowledge structural and functional similarities between phenomena across the religious-secular binary, allowing us to place questions of power, property, and space at the centre of our analysis, instead of focusing on the tedious question of whether text X or social phenomenon Y qualifies as religious and, correspondingly, which academic discipline ‘owns' it.

But the question remains: are ‘utopia’ and ‘utopianism’ useful second-order concepts for cross-cultural comparative projects? In other words: do they travel well? In my opinion, the answer to these questions is yes. Although none of the articles in this special issue focus on cases from Asia, there are numerous examples of utopian texts, imagery, and protest movements throughout the continent, only some of which are classified as religious. In this essay, I will provide a few such examples. Furthermore, I argue that East Asian cases are not merely useful for proving that ‘utopia’ is valuable as a comparative and analytical concept beyond the West; they may also provide relevant new insights into the nature of utopianism in general. In other words, East Asian utopianisms may raise new questions and provide a base for theorising, which can potentially shed new light on utopian ideas and practices elsewhere, including Europe. To illustrate this point, in this essay, I highlight two features of utopianism that merit further inquiry: its dependency upon theodicy and its ritual character. But let us first have a look at one particular utopian space: the city of Tenri in central Japan.

## A perfect city?

**Figure 1.**
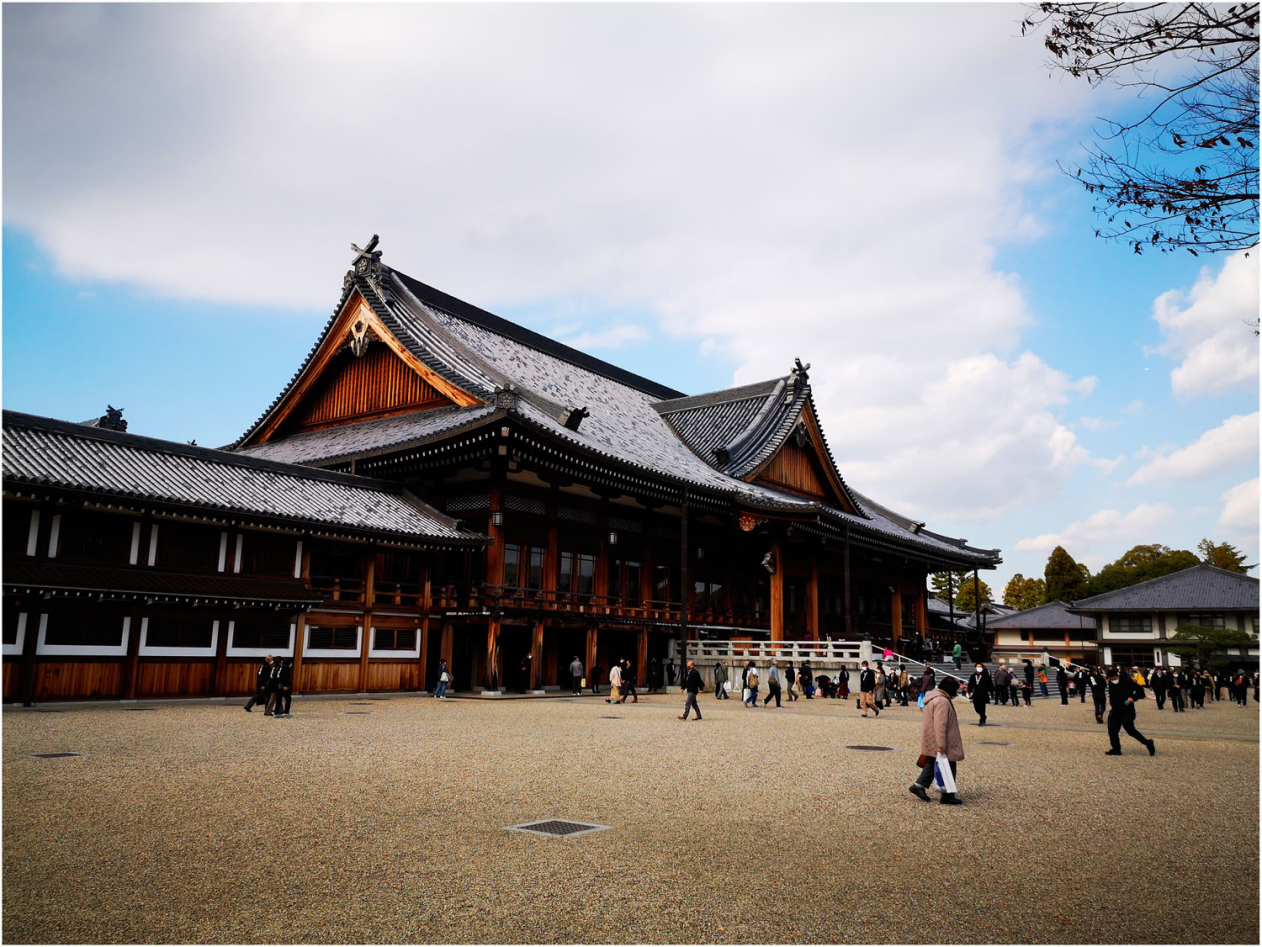
Main sanctuary (*shinden*) of the Tenrikyō Church Headquarters. Tenri City, Japan. Photo courtesy of Erica Baffelli.

The Japanese prefecture of Nara is rich in historic sites. It is home to ancient capitals, to Shinto shrines and sacred mountains that feature prominently in classical mythology and modern nationalist ideology (Rots 2017, 107–111; Shimizu 2022, 29–56), and to the oldest and most impressive Buddhist temple complexes in the country, several of which are listed as UNESCO World Heritage Sites. It is not only home to ancient shrines and temples, however, but also to various so-called new religious movements. Arguably one of the most impressive religious sites in the entire prefecture is the Tenrikyō Church Headquarters, the buildings of which were constructed in the late nineteenth and twentieth centuries (see Figure 1). According to Tenrikyō teachings, it is here, in the centre of the main sanctuary (*shinden*), that we find the *kanrodai*: the pillar that marks the location of the *jiba*, the place where God the Parent (Tenri Ō-no-mikoto) created humans and, consequently, the most sacred place in the world (Baffelli 2023, 199–201).

Tenrikyō traces its origins to the commencement of Nakayama Miki's (1798–1887) teachings in 1838. Nakayama was a local famer's wife who claimed to have been chosen by God the Parent to become the living ‘shrine of God’ (*kami no yashiro*), whose task it was to transmit God's message of salvation to humankind. She recorded her teachings in several sacred texts, the best-known of which is the *Ofudesaki*, which was compiled between 1869 and 1882. As Shimazono Susumu has pointed out, the *Ofudesaki* is characterised by a ‘combination of millennialism and chauvinism’ that is representative of the religious climate in the second half of the 19th century, ‘imply[ing] criticism of the political reforms and economic instabilities around the time of the Meiji Restoration that made farmers' lives desperate’ (Shimazono 2004, 150). As such, it is hardly unique: millenarian notions of divinely ordained socio-political change and corresponding calls for revolutionary action have been a recurring theme in early modern, modern, and post-war Japanese religious ideology. Examples are plentiful, ranging from the belief in divine catfish gods causing earthquakes and ‘world renewal’ (*yonaoshi*) in the late 18th century (Miura 2019) to Buddhist and Christian nationalist-utopian visions of the Japanese empire as a divine entity in the 1930s (Godart 2015; Rots 2010), and from the ‘human revolution’ promised by the lay Buddhist movement Sōka Gakkai (McLaughlin 2019) to the more violent apocalyptic visions of groups like Aum Shinrikyō and Kōfuku no Kagaku at the end of the 20th century (Baffelli and Reader 2012). Utopian and millenarian movements are and have long been part and parcel of the Japanese religious landscape, providing social and soteriological alternatives to the mainstream Buddhist, Shinto, and Christian institutions, which are generally more focused on preserving the status quo (Rots 2021).

Thus, what is special about Tenrikyō is not so much its utopian orientation, but rather the fact that it has been unusually successful in making its utopia take shape physically; in other words, in utopian place-making on the ground. How does one make a perfect place in an imperfect world? As pointed out by Beutter and Sewordor in their article in this special issue, this is the classical conundrum of utopianism: the moment the utopia becomes a real thing in the world, it ceases to be perfect, for the simple reason that the world is an imperfect, messy, and unpredictable place that is impossible to control. Utopian communities can never be fully isolated and self-sustaining, no matter how hard they try – there are always interactions with the surrounding environment, society, and state. This is the tragic fate of all utopias, illustrated compellingly in M. Night Shyamalan's classic thriller *The Village* (2004): isolation is impossible and corruption inevitable, so the utopia is bound to fail. There is no such thing as a perfectly bounded community, just as there can be no fully autonomous person. All bodies, collective or individual, are porous; all are shaped by and depend upon numerous other bodies with which they are entangled.

Nevertheless, Tenrikyō has made considerable efforts to make their perfect society come alive, building an entire city around the *jiba* to house their community and accommodate pilgrims visiting from elsewhere, while also serving as a model of a good urban society for the rest of humanity: Tenri City. This city, which today has a population of around 67,000, was created in 1954 after a round of municipal mergers. As Erica Baffelli has pointed out, ‘while the decision for the merger was based on administrative decisions, the presence of Tenrikyō played an important role in its development and its naming, making Tenri City the only city in Japan named after a religious organisation’ (Baffelli 2023, 202). Unsurprisingly, the Tenrikyō Church Headquarters occupy a prominent place in the city centre, as do other buildings associated with the religion, such as pilgrim lodgings, a museum, schools, and Tenri University. But Tenri is not a city exclusively for Tenrikyō followers, and it is not merely a religious centre, either. Like other Japanese cities, it has a train station, shops, a concert hall, restaurants, and even Buddhist temples. At first sight, Tenri comes across as a typical post-war Japanese suburban space, built around a covered shopping street (*shōtengai*) housing various small family enterprises, and residential areas consisting of low blocks that are divided into small apartments (*manshon*). Although framed in religious terms at the time of its construction (Baffelli 2023, 204), the shape and outline of the city in fact reflect the dominant early post-war urban planning paradigm. Today, this cityscape comes across as outdated, and Tenri has not escaped the fate of other small- to medium-sized Japanese cities, towns, and suburban neighbourhoods: an aging population, economic hardship, and the decline of the *shōtengai* (Baffelli 2023, 207).

How, then, is the utopian space different from any other Japanese urban space? How does Tenrikyō preserve its ideal urban society as a lived reality, not merely an unattainable fantasy? The answer, I believe, lies in a combination of theodicy and ritualised action. Every utopianism that exists not merely as a literal trope but as a lived social practice needs both. It needs theodicy to cope with the aforementioned conundrum that absolute perfection is impossible, and perhaps even undesirable, as it would put an end to the constant living towards the future that constitutes one of lived utopianism's defining features. And it needs ritualised, embodied action in order to stay alive as a social practice that can shape identities and mobilise people.

## Theodicy

Roughly speaking, the articles in this special issue can be divided into two groups: those that discuss utopian *imagery* in literary, academic, and popular scientific texts (Hermann, Krüger, Mignon, and Rota) and those that focus primarily on utopianism as a *social practice* aiming to create the perfect society (Beutter and Sewordor, Dresen, and Kirsch). Both types of utopianism have to address the question of theodicy; in other words, how to make sense of imperfection in a perfect world. In the case of utopian imagery in literature or science, this need not be a problem, because the utopian ideal promises the erasure of human suffering, and as long as it remains a fantasy, it will not have to face the reality that violence, pain, death, and mourning are intrinsic features of life on this planet. Indeed, it is the very contrast between this reality and the utopian imagination that makes the fantasy attractive. But as soon as people seek to bring utopian ideals into practice – as soon as the utopianism ceases to be an other-worldly fantasy and becomes a this-worldly mobilisation force, in other words – the inescapability of imperfection and suffering becomes an urgent problem. This comes to the fore most clearly in the articles of Beutter and Sewordor, on a Christian utopian community as a ‘lived social project’ in Ghana, and Kirsch, on a nineteenth-century ‘vernacular utopia’ in the US. Both articles provide interesting examples of the discursive strategies employed by community leaders to explain and sanction undesirable emotions and behaviour, preventing such inconvenient realities from undermining the larger utopian project.

Utopian ideologies and movements are always future-oriented, even if the blueprint for the perfect future society is located in a mythical past. This applies to secular utopianisms such as communism and radical nationalism (Levinger and Lytle 2001) as much as to millenarian movements that seek to pave the way for the (first or second) coming of their Messiah. As Beutter writes in her article, utopia can be defined ‘as a positive and collective vision of an ideal future that transcends a negative present state […] and utopian thinking and action as an aspirational orientation to go beyond an imperfect *is* to implement an idealized *ought*.’ As implied by the verb ‘ought’, perfection has not yet been achieved, but it is attainable in the near future. Even movements that have succeeded in creating a demarcated, ‘utopian’ physical space for their community, such as Salem in Ghana or Tenri in Japan, are oriented towards a future in which their not-yet-completely-fulfilled vision will finally become reality. Theodicy is thus entwined with temporality; the promise of future gratification goes hand in hand with a profound rejection of the presumed causes of its delay, whether moral, spiritual, ethnic, or material.

According to Tenrikyō teachings, the perfect harmonious society has not yet come to be, even if some important steps have been made. The cause of the delay, in this case, is pollution. Tenrikyō has adopted the popular Shinto notion that misfortune and pain are caused by pollution (*kegare*) and that purification rituals are needed to restore balance. Within mainstream Shinto, this is the natural order of things; pollution is caused by perfectly normal things such as blood and death, which may provoke divine discomfort and wrath and thereby cause calamities, but which can be removed through ritual action, if only temporarily. Thus, mainstream Shinto shrine rituals are not utopian; they have a profoundly this-worldly orientation. In Tenrikyō and similar ‘new’ religious movements, however, the pollution is not merely situational, and not part of the natural order of things; it is an existential and moral threat that prevents the perfect society from taking place. In other words: it is part of what *is*, but not what *ought to be*. This pollution is not merely an abstract, spiritual notion, but a physical reality. Dirt is the root cause of evil. This is why, when walking in the shopping street of Tenri, one constantly comes across shopkeepers who are dusting off their groceries and goods, even though there is no dust to be seen. This is why, in the Tenrikyō headquarters, it is common to see young members moving around on their knees, mopping the floors until they shine. And this is why the public toilets here are always spotlessly clean. Dirty toilets are not just a nuisance; they undermine the utopian project. Thus, pollution serves as theodicy, explaining why the utopian project has not yet reached its fulfilment. In this case, the explanation is material as well as spiritual. And, crucially, the solution lies not merely in moral behaviour or the cognitive acceptance of a particular soteriology; it lies in ritual action.

## Ritual action as utopian action

Not all ritual action takes place in the main hall of a temple or church. In fact, much important ritual labour takes place behind the scenes. Much of this ritual labour is conducted by women, and much of it has escaped the eyes of anthropologists and historians of religion, who tend to focus on what happens in front of the main altar, not in the kitchen. But as we have seen, cleaning toilets can be a ritual act that pleases the gods and brings merit to the people performing it (Reader 2005). And no temple festival can take place without the labour of mostly female volunteers who prepare food offerings.

In a fascinating recent article (Puett 2019), Michael Puett introduces ritual theories from ancient China and applies them to debates on the use of indigenous terminology within contemporary anthropology. Puett distinguishes between different types of ritual action. On the one hand, there are rituals that serve to *domesticate*. Just like agriculture consists of techniques to domesticate non-human animals and plants and make them controllable, ritual serves to bring non-human entities into the realm of society: ‘what was before a dangerous (for humans) world of highly capricious spirits and potentially malevolent ghosts is transformed into a world of benevolent spirits and ancestors acting in support of humanity’ (Puett 2019, 443). Ritual is not a one-time event, but a never-ending labour. This type of ritual is grounded in a view of the world as imperfect, fragmented, and constantly in need of ritual action to cultivate and preserve the social-natural order of things, so that it does not fall apart. In other words, it is a fundamentally non-utopian, this-worldly ontology: the world is a dangerous place, and ritual helps for keeping things in place and getting some protection, but only for a while. Ritual is what prevents society from falling apart, but there is no such thing as perfection. In fact, perfection may not even be desirable:

The transformations, in other words, are temporary and inadequate. Ghosts and spirits are always more powerful than the ritual attempts to domesticate them. They always exceed the rituals. And when the rituals fail, the ghosts return. […] Despite human attempts at domestication, the world continues to be disparate and dominated by capricious and highly dangerous powers (Puett 2019, 444).

On the other hand, ancient China was also home to movements that did *not* seek to domesticate spirits in order to preserve the societal status quo; movements that, by contrast, wished to create or ‘restore’ a perfectly harmonious society. According to them,

The world of dangerous ghosts […] is not an inherent part of the cosmos that humans are trying to alter through their work of domestication. The cosmos on the contrary is inherently harmonious, and proper human behavior plays a crucial role in maintaining that harmony. The dominance of a world of dangerous ghosts is purely a result of improper human activity (Puett 2019, 453).

These movements were utopian and millenarian in the sense that they used their vision of a perfectly harmonious past society as a blueprint for the future – and, as such, diametrically opposed to the aforementioned view of the world as a dangerous place. They, too, developed rituals, but their rituals were focused on so-called self-divinisation, where the goal is ‘not to domesticate the divine but rather to divinize the human’ (Puett 2019, 451).

Can we conceive of utopian action as ritual action in this second sense, not as a type of *domestication* of a dangerous other in an inherently chaotic world, but as acts of *cultivation* of the self and the collective? Arguably, most institutionalised religions, state agencies, political parties, and corporate entities are preservationist: they seek to preserve the political and economic system. They may want to fix some cracks in the system, but they do not wish to overthrow it. Utopian movements, by contrast, want to replace the current, broken system by something altogether different, and ritual action may be a way to achieve that goal. In the case of Tenrikyō, this self-cultivation takes the shape of ritualised cleaning, as well as bodily movements (a so-called ‘hand dance’, *te-odori*). Less appealingly: the utopian violence of radical Maoist organisations like the Khmer Rouge, for instance, was highly ritualised (Delano and David Knottnerus 2018). And what if we think of the disruptive action of Extinction Rebellion not merely as mediatised performances that serve to get public attention for the urgency of climate action, but as self-cultivation practices that seek to create a better world through ritual action? Radical environmentalism arguably constitutes a type of utopianism, combining millenarian notions of temporality and rupture with ritual cultivation practices (Pike 2017). This does *not* mean that Extinction Rebellion and likeminded groups should be classified as ‘religious’; many environmentalists have an ambivalent or even antagonistic attitude to institutionalised religion and its symbols and practices, and I disagree with a scholar like Bron Taylor who uses ‘dark green religion’ as a lazy umbrella term for all types of moral and ritual engagement with non-human Others (Taylor 2009). It *does* mean that there are some structural similarities between radical environmentalist groups, millenarian religions, and other utopian social movements (e.g., communist or fascist), not only in terms of temporal ideology and theodicy, but also, possibly, in terms of ritual action. This, at least, is a topic that merits further investigation. That is, the relationship between ritual and utopia needs to be explored further if we want to understand ‘utopia’ not merely as a discursive construct but also as a social practice. Put differently: to what extent is vernacular utopianism ritualised?

## Conclusion

The concepts ‘utopia’ and ‘utopianism’ are useful heuristic tools for understanding a particular genre of texts and set of social practices. What these texts and practices have in common is their *temporal* and *moral* dimensions. They are future-oriented, projecting their vision of the perfect society onto a future that is either imminent or faraway, but always in the not-yet, even if the first steps towards its realisation have been taken. And they have a strong ethical orientation that is directly connected to their temporality, typically rejecting present-day society on moral grounds and promising a better world. If communicated convincingly and compellingly, such visions can have profound mobilising potential. This applies to millenarian Pentecostalism, militant Zionism, and some Japanese new religions as much as to purportedly secular ideologies such as Maoism-Leninism, some types of ethnic nationalism (Levinger and Lytle 2001), and certain currents of environmentalist thought (Kowalewski 2023). And this is exactly why these terms can be fruitfully employed as comparative second-order (i.e., metalanguage) concepts within the study of religion\s: they can help us see similarities between texts and movements that are not usually studied in conjunction, which may shed new light on the texts and movements in question.

The articles in this special issue thus do important ground-breaking work. The volume brings together case studies that appear very different thematically and methodologically, discussing disparate topics ranging from late Ottoman literature to late-Soviet Baltic religious movements, and from contemporary transhumanist techno-fantasies to a Christian mission organisation in Africa. It is precisely the shared conceptual framework that binds these different cases together. Reading the articles in conjunction leads to interesting new comparative insights.

As I have argued in this response, the conceptual and theoretical interventions put forward in this special issue can be developed further by bringing them in dialogue with cases and theories from East Asia. I have introduced the case of Tenri City in Japan, which is interesting because it constitutes an example of a ‘vernacular utopia’ that is a real physical place shaped in accordance with utopian ideals, even if true perfection has turned out to be unattainable. Drawing upon this case, I have argued that, like ‘utopia’, ‘theodicy’ should be reconsidered as a useful analytical concept for our discipline, and that theodicy is a core component of utopian thought and action that can and should be studied comparatively. And I have suggested that utopianism can be fruitfully studied not only as an ideological construct or literary genre, but also as a social practice that includes a particular type of ritual action. Michael Puett's work on ritual theory from ancient China is highly relevant in this regard, especially his distinction between two types of ritual logic: *ritual as domestication* in an imperfect, non-utopian world, and *ritual as self-divinisation* that is grounded in notions of lost harmony and has a profound utopian orientation. Arguably, this distinction is relevant not only in ancient China, but also in today's world. It is helpful not only for analysing practices and ideas that are classified as religious, but also for understanding other types of utopian action, including radical environmentalism. In any case, the relationship between ritual action and utopian action needs further inquiry and theorisation.
